# Comparative Physiological and Transcriptome Analyses Reveal Mechanisms of Salicylic-Acid-Reduced Postharvest Ripening in ‘Hosui’ Pears (*Pyrus pyrifolia* Nakai)

**DOI:** 10.3390/plants12193429

**Published:** 2023-09-28

**Authors:** Jing Zhang, Mengmeng Wen, Rong Dai, Xiao Liu, Chunlei Wang

**Affiliations:** College of Horticulture and Landscape Architecture, International Research Laboratory of Agriculture and Agri-Product Safety, Key Laboratory of Plant Functional Genomics of the Ministry of Education, Yangzhou University, 48 Wenhui East Road, Yangzhou 225009, China; zhangj45@yzu.edu.cn (J.Z.); wenmm1014@163.com (M.W.); dairong1100@163.com (R.D.); liuxiao@yzu.edu.cn (X.L.)

**Keywords:** sand pear, salicylic acid, fruit ripening, transcriptome

## Abstract

Postharvest ripening of sand pear fruit leads to quality deterioration, including changes in texture, flavor, and fruit color. Salicylic acid (SA), an important defense-related hormone, delays fruit ripening and maintains fruit quality, but the underling mechanism remains unclear. Herein, we evaluated the efficacy of SA in delaying the ripening process of *Pyrus pyrifolia* cv. ’Hosui’ pear fruit, as evidenced by the reduction in fruit weight loss, inhibition of firmness loss, cell wall degradation and soluble sugars, and retention of total phenols. Based on comparative transcriptomic data, a total of 3837 and 1387 differentially expressed genes (DEGs) were identified during room-temperature storage of control fruit and between SA-treated and control fruit, respectively. Further KEGG analysis revealed that the DEGs were mainly implicated in plant hormone signal transduction, starch and sugar metabolism, and cell wall modification. Moreover, exogenous SA treatment also altered the expression of many transcription factor (TF) families, including those in the ethylene-responsive factor (ERF), NAM, ATAF, CUC (NAC), basic helix-loop-helix (bHLH), basic leucine zipper (bZIP), and v-myb avian myeloblastosis viral oncogene homolog (MYB) families. Together, the results offer important insights into the role of SA-responsive genes in controlling fruit ripening in sand pears.

## 1. Introduction

Pears (*Pyrus* spp.) are an economically important fruit crop with a juicy taste and rich nutrition, and they are widely planted around the world [1], with an annual production of 25.66 million tons in 2021 (FAOSTAT). Presently, pear cultivars are divided into five species, including *P. communis*, *P. pyrifolia*, *P. bretschneideri*, *P. sinkiangensis*, and *P. ussuriensis* [2]. Of these, *P. pyrifolia*—the Japanese pear or sand pear—is a major species of cultivated pear that is mainly distributed in China, Japan, and Korea. To ensure a year-round supply and extend the shelf life of sand pear fruits, they are typically not consumed immediately after harvest. Instead, most of the harvested pear fruits undergo a more or less prolonged postharvest period where they are stored and then supplied to the market at the appropriate time. However, fruit storage potential is closely related to the maximum level of ethylene production [3]. Ethylene production in different sand pear cultivars varies during fruit ripening, suggesting that there are both climacteric and non-climacteric types [4,5,6]. In typical climacteric sand pear cultivars, rapid postharvest ripening progression that is regulated by ethylene accelerates ripening-related characteristics such as water loss, flesh softening, browning, and decay, resulting in fruit quality deterioration and reduced shelf life [7,8]. Furthermore, sand pear fruits predominantly ripen during the mid-summer season, and continuous exposure to high temperatures and undesirable storage conditions can hasten tissue disintegration and cell death [9,10]. Hence, developing efficient storage techniques to maintain postharvest quality properties are of great significance in sand pear fruit industrialization. In past decades, several treatment methods, including low temperature, 1-methylcyclopropene (1-MCP), modified atmosphere packaging, phytohormones, and edible coatings, have been utilized on pears (including several sand pear cultivars) to extend their postharvest storability and minimize their deterioration [9,10,11,12,13,14].

Fruit ripening is the final stage of fruit development. Fleshy fruit undergoes a genetically programmed and developmentally regulated postharvest ripening process that involves various physiological and biochemical changes, such as discoloration, texture changes, flavor loss, and nutritional value reduction, which significantly reduce fruit quality and consumer acceptance [15,16,17]. In pears, softening changes like reductions in firmness, cell wall degradation, and decreases in cell turgor pressure have been described as key physiological processes during postharvest ripening [4,6]. Fruit softening is closely related to the degradation and remodeling of primary cell walls that are composed of cellulose, hemicellulose, and pectin [18,19]. Pectin is the main component of the glue layer in the primary cell wall, and different forms of pectin, such as water-soluble pectin (WSP), ionic soluble pectin (ISP), and covalent binding pectin (CSP), can be extracted in different ways [20]. In the process of pear fruit’s softening, the contents of cellulose, hemicellulose, and CSP usually decrease, while the abundance of WSP increases [14,21,22]. As for fruit flavor, the composition and amounts of soluble sugars (including sucrose, glucose, fructose, and sorbitol) play key roles in determining the sweetness of pear fruit [23]. During postharvest pear storage, significant changes were observed in individual soluble sugars [23,24,25]. To date, several ripening-related genes have been identified in pears, including those correlated with cell wall metabolism (e.g., *polygalacturonase 1/2* (*PG1/2*) from *P. pyrifolia* cv. ‘Nijisseiki’ and ‘Starkrimson’; *pectin methylesterase 2/3* (*PME2/3*), *pectin lyase* (*PL*), *β-galactoside 1/2/4* (*β-gal1*/2/4), and *α-arabinofuranase 1* (*ARF1*) from *P. communis* cv. ‘Docteur Jules Guyot’) [8,14,21,22]; sweetness (*acid invertase 1* (*Ac-Inv1*), *invertase inhibitor 5* (*II5*) and *sucrose phosphate synthase 1* (*SPS1*) from *P. pyrifolia* cv. ‘Hosui’; and *neutral invertase* (*NI*), *sucrose synthase* (*SUS*), and *SPS* from *Pyrus ussuriensis* cv. ‘Nanguo’) [23,24,25].

SA is a natural hormone that is widely distributed in plants and plays important roles in plants’ growth, development, and responses to biotic and abiotic stresses [26]. In addition, several studies have shown that treatments with exogenous SA or its derivatives (acetylsalicylate (ASA) and methyl salicylate (MeSA)) can maintain fruit quality and extend shelf life by decreasing ethylene production and respiration rates, activating the antioxidant system and regulating the metabolism of cell wall components, sugars, acids, and aromatic volatiles of the fruit [27,28,29,30]. For instance, SA delayed tomato and kiwifruit ripening by suppressing the expression levels of 1-aminocyclopropane-1-carboxylic acid oxidase (ACO) and 1-aminocyclopropane-1-carboxylic acid synthase (ACS) and preventing the conversion of 1-aminocyclopropane-1-carboxylic acid (ACC) to ethylene, which is the most important plant hormone responsible for fruit ripening [31,32]. Exogenous SA enhanced the expression levels of *PpLOX*, *PpHPL*, *PpADH*, and *PpAAT* in peaches (*Prunus persica* L. Batsch., cv. ‘Hujingmilu’) during cold storage and increased the amounts of fruity-note volatile esters and lactones [33]. To date, several studies on pear fruit have revealed that SA treatment with optimal concentrations could also be effective in alleviating chilling injury, maintaining postharvest quality, and reducing fruit decay and tissue browning [34,35,36,37]. However, the molecular mechanism underlying SA-alleviated pear fruit ripening is less known.

‘Hosui’ (*Pyrus pyrifolia* Nakai) is an important sand pear cultivar with high yield, high pest-resistance capacity, and good consumption quality, and is widely planted in China [12]. As a typical climacteric fruit, ‘Hosui’ pears exhibit rapid increases in respiratory rate and ethylene production during postharvest storage, accompanied by severe fruit quality deterioration [38], and they were selected for this study. In the present paper, the influences of SA on maintaining fruit quality and delaying ripening of ‘Hosui’ pear fruit under room-temperature storage were analyzed, and RNA sequencing (RNA-Seq) was conducted to reveal the molecular mechanisms underlying SA-mediated preservation of sand pear fruit. These results offer important insights into the role of SA-responsive genes in controlling fruit ripening.

## 2. Results

### 2.1. Effect of SA Treatment on the Postharvest Ripening Process of Pear Fruit

**Figure 1:**
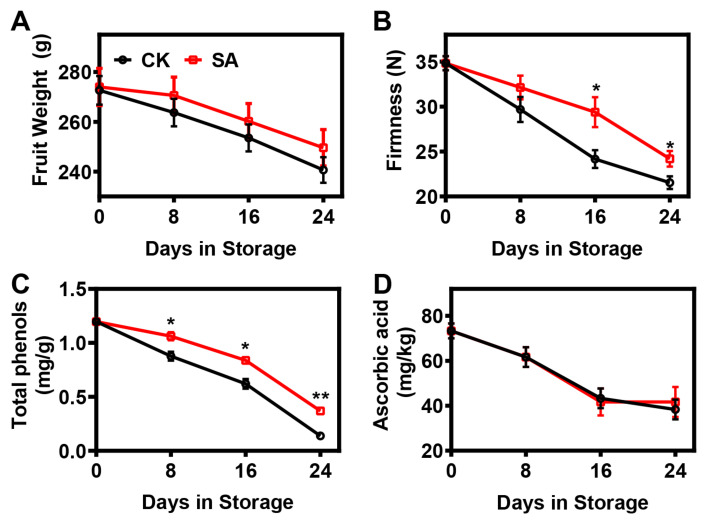
Physiological changes in pear fruit treated with sterile water (CK) and SA during room-temperature storage: (**A**) Fruit weight. (**B**) Firmness. (**C**) Total phenols. (**D**) Ascorbic acid. Error bars indicate SEs from 15 (for fruit weight), 9 (for firmness), and 3 (for total phenols and ascorbic acid) replicates. The statistical analysis was performed using two-tailed Student’s *t*-tests. The asterisks indicate significant differences: * *p* < 0.1; ** *p* < 0.01.

In this study, ‘Hosui’ pear fruits’ weight and firmness changes during postharvest ripening are shown in Figure 1. The fruit weight of ‘Hosui’ pears continuously decreased throughout the whole storage period, regardless of treatment, but SA treatment alleviated the reduction in fruit weight (Figure 1A). Consistent with the pear fruit weight trend, fruit firmness declined throughout the storage period, and the softening trajectory was retarded by SA treatment (Figure 1B).

Total phenols and ascorbic acid are important antioxidant components that are tightly associated with tissue damage and fruit aging [39]. In this study, the total phenols and ascorbic acid in ‘Hosui’ pear fruit presented decreasing tendencies during postharvest storage (Figure 1C,D). The trend of total phenols was significantly mitigated by SA treatment (Figure 1C). However, no significant difference in ascorbic acid was observed between the control and SA-treated pear fruit (Figure 1D).

### 2.2. Effect of SA Treatment on the Cell Wall Composition of Pear Fruit during Room-Temperature Storage

**Figure 2:**
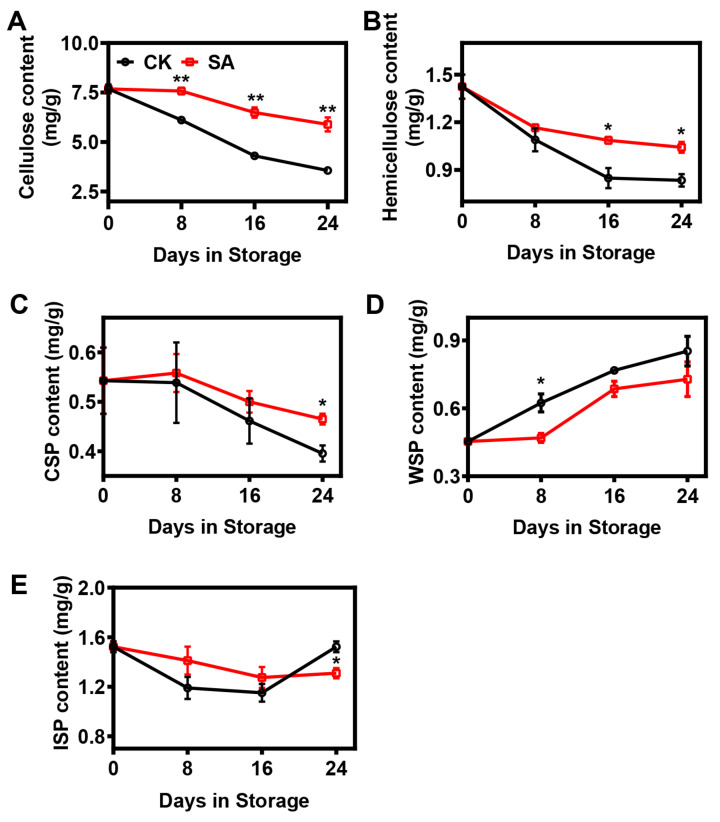
Effects of SA treatment on the cell wall material (CWM) composition of pear fruit during room-temperature storage: (**A**) Cellulose content. (**B**) Hemicellulose content. (**C**) CSP content. (**D**) WSP content. (**E**) ISP content. Error bars indicate SEs from 3 replicates. The statistical analysis was performed using two-tailed Student’s *t*-tests. The asterisks indicate significant differences: * *p* < 0.1; ** *p* < 0.01.

In this research, statistical analysis demonstrated that the cellulose, hemicellulose, and CSP contents continuously decreased during the postharvest storage period, while the rate of decrease was significantly inhibited in SA-treated pear fruit (Figure 2). At the 24th day of storage, the cellulose contents fell to 47% and 77%, the hemicellulose contents dropped to 59% and 73%, and the CSP contents decreased to 73% and 86% of the levels measured on day 0 for the control and SA-treated pear fruit, respectively (Figure 2). In contrast, the contents of WSP increased remarkably during postharvest storage, and the level was lower in SA-treated pear fruit (Figure 2D). Furthermore, the ISP contents in the two groups fluctuated during postharvest storage, and the ISP content in the control pear fruit was significantly higher at the 24th day of storage compared with the SA-treated pear fruit (Figure 2E).

### 2.3. Effect of SA Treatment on the Soluble Sugar Composition of Pear Fruit during Room-Temperature Storage

**Figure 3:**
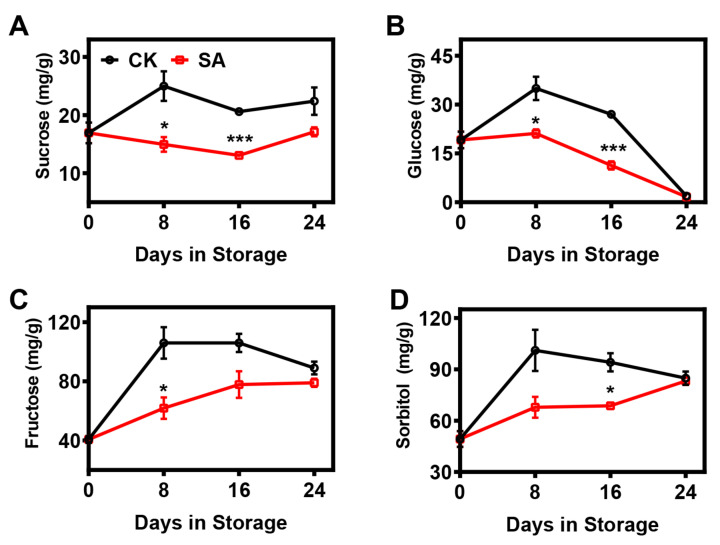
Effects of SA treatment on the soluble sugar composition of pear fruit during room-temperature storage: (**A**) Sucrose content. (**B**) Glucose content. (**C**) Fructose content. (**D**) Sorbitol content. Error bars indicate SEs from 3 replicates. The statistical analysis was performed using two-tailed Student’s *t*-tests. The asterisks indicate significant differences: * *p* < 0.1; *** *p* < 0.001.

Here, the contents of soluble sugars were analyzed throughout postharvest storage. In control fruit, the contents of sucrose, fructose, and sorbitol tended to increase during the first 8 d and then remained steady, while the glucose content first increased and then decreased progressively (Figure 3). In SA-treated fruit, the changes in sugar contents during storage were strongly affected relative to those in the controls. The level of glucose in SA-treated fruit gradually declined after the 8th day of storage, while a reverse phenomenon was observed for fructose and sorbitol; on the other hand, the sucrose content remained relatively constant throughout storage (Figure 3). Overall, SA treatment strongly diminished the soluble sugar contents compared with the controls (Figure 3).

### 2.4. Characterization of Transcriptional Changes of Pear Fruit in Response to Salicylic Acid by RNA-Seq

**Figure 4:**
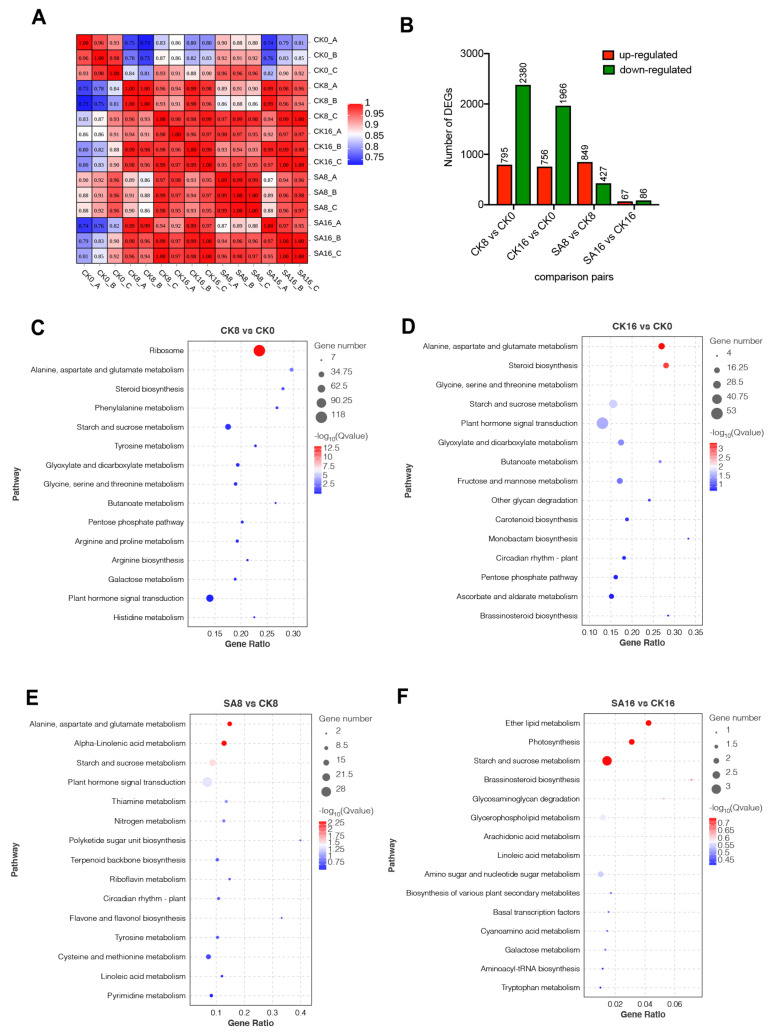
Identification and expression analysis of the DEGs involved in pear fruit ripening and SA responses: (**A**) Correlation analysis between the fifteen samples (CK 0, 8, 16 d and SA 8, 16 d, with three biological replicates). (**B**) Comparison of the DEGs among different comparison groups and the numbers of up− and downregulated DEGs in the individual comparison groups. (**C**,**D**) KEGG pathway analysis of DEGs during pear fruit ripening at 8 d (**C**) and 16 d (**D**) compared to 0 d after treatment for the control group. (**E**,**F**) KEGG pathway analysis of DEGs between pairwise comparisons of SA treatment and the control group at 8 d (**E**) and 16 d (**F**). Gene ratio represents the ratio of the number of significantly expressed genes in a particular pathway to the total number of genes in the pathway.

To identify key genes contributing to the observed physiological traits, and to clarify the potential metabolism in response to exogenous SA treatment, we performed a comparative RNA-Seq analysis. As pear fruit exhibited the most significant physiological changes during 0–16 days of storage, pear fruit samples, including CK (0, 8, 16) and SA (8, 16), were selected for RNA extraction and RNA-Seq analysis, with three biological replicates. The details regarding the sequencing data for each sample are listed in Table S1. An average of 6.95 billion clean reads were obtained per library, of which approximately 77.65% were mapped to the pear genome (Table S1). The RNA-Seq results indicated that the three biological replicates were highly correlated (R^2^ ≥ 0.93), and the samples at different timepoints could be clearly distinguished (0.97 ≥ R^2^ ≥ 0.81) (Figure 4A). Using the criteria listed in the Materials and Methods section, a total of 4308 DEGs were identified among the following four comparison groups: CK8, CK16 vs. CK0, SA8 vs. CK8, and SA16 vs. CK16 (Figure 4B, Table S2). Of these, 3175 DEGs (including 795 upregulated and 2380 downregulated) and 2722 DEGs (including 756 upregulated and 1966 downregulated) appeared at the 8th day and the 16th day compared to day 0 after treatment for the control group, respectively. Meanwhile, SA treatment induced dramatic changes in the transcript abundances of pear fruit, including 1276 and 153 DEGs identified in fruit treated with SA at the 8th day and the 16th day, respectively, compared to non-treated pear fruits (Figure 4B).

A KEGG enrichment analysis showed that ‘Ribosome’, ‘Plant hormone signal transduction’, and ‘Starch and sucrose metabolism’ were enriched in the CK8 vs. CK0 group (Figure 4C). In addition, ‘Alanine, aspartate and glutamate metabolism’, ‘steroid biosynthesis’, ‘Starch and sucrose metabolism’, and ‘Plant hormone signal transduction’ were significantly enriched in the CK16 vs. CK0 group (Figure 4D). For exogenous SA treatment, ‘Alanine, aspartate and glutamate metabolism’, ‘Alpha-Linolenic acid metabolism’, ‘Starch and sucrose metabolism’, and ‘Plant hormone signal transduction’ were the most enriched pathways on the 8th day compared with the control group (Figure 4E). ‘Ether lipid metabolism’, ‘Photosynthesis’, and ‘Starch and sucrose metabolism’ were the most enriched pathways on the 16th day (Figure 4F). It can be seen from the KEGG analysis that ‘plant hormone signal transduction’ and ‘starch and sucrose metabolism’ were significantly enriched in both pear fruit ripening and exogenous SA treatment (Figure 4C–F), indicating that plant hormone signal transduction and sugar transformation may play important roles in SA-reduced pear fruit ripening.

### 2.5. DEGs Related to Plant Hormone Signal Transduction

**Figure 5:**
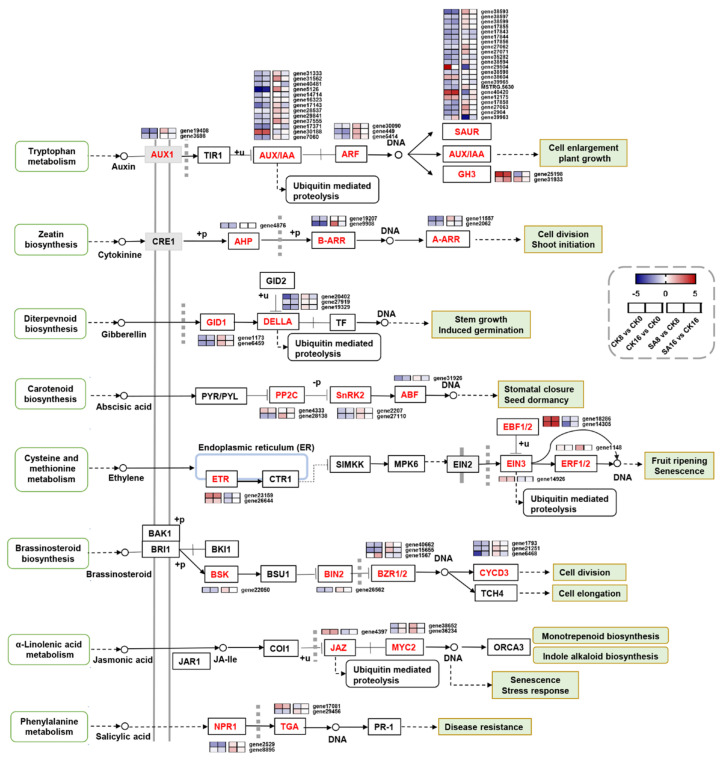
Expression profiles of DEGs associated with different plant hormones’ signal transduction. Gradient colors represent the log_2_ fold change in gene expression between different times and treatments. The red and blue colors indicate the up− and downregulated genes, respectively.

To obtain a deeper understanding of the effect of SA treatment on plant hormone signal transduction during pear fruit ripening, the expression patterns of 78 DEGs were annotated to different plant hormone signal transduction pathways. As shown in Figure 5, the auxin signal transduction pathway was remarkably affected during SA-reduced pear fruit ripening. A total of 42 genes mapped to the auxin signal transduction pathway, including 2 *AUX1* (auxin influx carrier) genes, 13 *AUX/IAA* (auxin responsive protein) genes, 3 *ARF* (auxin response factor) genes, 22 *SAUR* (SAUR family protein) genes, and 2 *GH3* (Gretchen Hagen 3) genes, of which 8 DEGs were upregulated and 31 DEGs were downregulated in the CK8 vs. CK0 and CK16 vs. CK0 comparison groups, while 14 DEGs were upregulated and 9 DEGs were downregulated in the SA8 vs. CK8 and SA16 vs. CK16 comparison groups (Figure 5 and Table S3). The brassinosteroid signal transduction pathway contained eight genes, including *BSK* (BR-signaling kinase), *BIN2* (brassinosteroid-insensitive 2), *BZR1/BES1* (brassinazole-resistant 1/BRI1-ethylmethylsulfone-suppressor 1), and *CYCD3* (cyclin D3, plant), of which one DEG was upregulated and seven DEGs were downregulated in the CK8 vs. CK0 and CK16 vs. CK0 comparison groups, while only one upregulated DEG was identified in the SA8 vs. CK8 comparison group (Figure 5, Table S3). Six genes were identified in the ethylene signal transduction pathway, including two genes annotated as ETR (ethylene receptor), one gene annotated as EIN3 (ethylene insensitive 3), two genes annotated as EBF2 (EIN3-binding F-box protein), and one gene annotated as ERF (TF), of which five DEGs were upregulated in the CK8 vs. CK0 and CK16 vs. CK0 comparison groups, whereas one DEG was upregulated and two DEGs were downregulated in the SA8 vs. CK8 and SA16 vs. CK16 comparison groups (Figure 5, Table S3). Additionally, most of the DEGs associated with particular signal transduction pathways, including the cytokinin, abscisic acid, gibberellin, and jasmonic acid signal transduction pathways, were significantly downregulated in pear fruit during postharvest ripening, and several of the identified DEGs, such as one *A-ARR* (two-component response regulator ARR-A family), one *B-ARR* (two-component response regulator ARR-B family), one *PP2C* (protein phosphatase 2C), two *GIDs* (gibberellin-insensitive dwarf), and two *MYC2s* (TF), were upregulated in SA-treated pear fruit (Figure 5, Table S3). The above results suggest that SA treatment has vital effects on plant hormone signal transduction during pear fruit ripening.

### 2.6. DEGs Related to Sugar Metabolism

**Figure 6:**
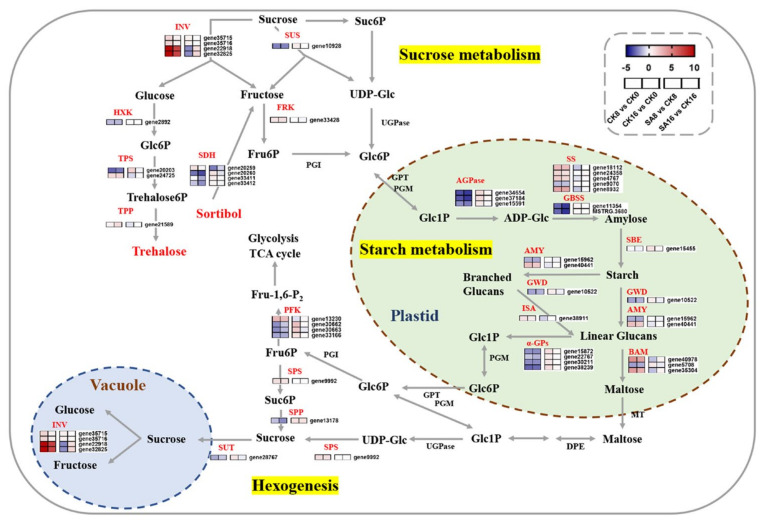
Expression profiles of DEGs associated with sugar metabolism. Gradient colors represent the log_2_ fold change in gene expression between different times and treatments. The red and blue colors indicate the up− and downregulated genes, respectively.

SA treatment dramatically reduced the accumulation of soluble sugars during postharvest ripening (Figure 3). Since DEGs were highly enriched in the ‘Starch and sucrose metabolism’ pathway (Figure 4), we further investigated the regulation of starch and sucrose metabolism pathways and identified 43 genes that were significantly differentially expressed (Figure 6 and Table S4). The expression levels of DEGs encoding invertase (INV), SPS, and fructokinase (FRK) were significantly upregulated in pear fruits undergoing postharvest ripening and were highly correlated with the changes in soluble sugars in control ‘Hosui’ fruit (Figure 6 and Table S4). Among them, two *INVs* (*gene22918* and *gene32825*) exhibited the most significant fold changes between the control and SA-treated pear fruit, reaching fivefold (Figure 6, Table S4). In contrast, some other key genes involved in soluble sugar biosynthesis showed the opposite expression pattern, including sucrose synthase (SUS), sucrose-phosphatase (SPP), sucrose transport protein (SUT), and hexokinase (HXK) (Figure 6). Additionally, the DEGs associated with starch synthesis and degradation, including ADP-glucose pyrophosphorylase (AGPase), starch synthase (SS), granule-bound starch synthase (GBSS), starch-branching enzyme (SBE), α-amylase (AMY), β-amylase (BAM), glucan water dikinase (GWD), isoamylase (ISA), and α-glucan phosphorylase (α-GP), exhibited differentiated expression patterns (Figure 6). These results suggest that changes in genes related to sugar synthesis and metabolism contribute to pear fruit ripening and exogenous SA treatment.

### 2.7. DEGs Related to Fruit Softening

**Figure 7:**
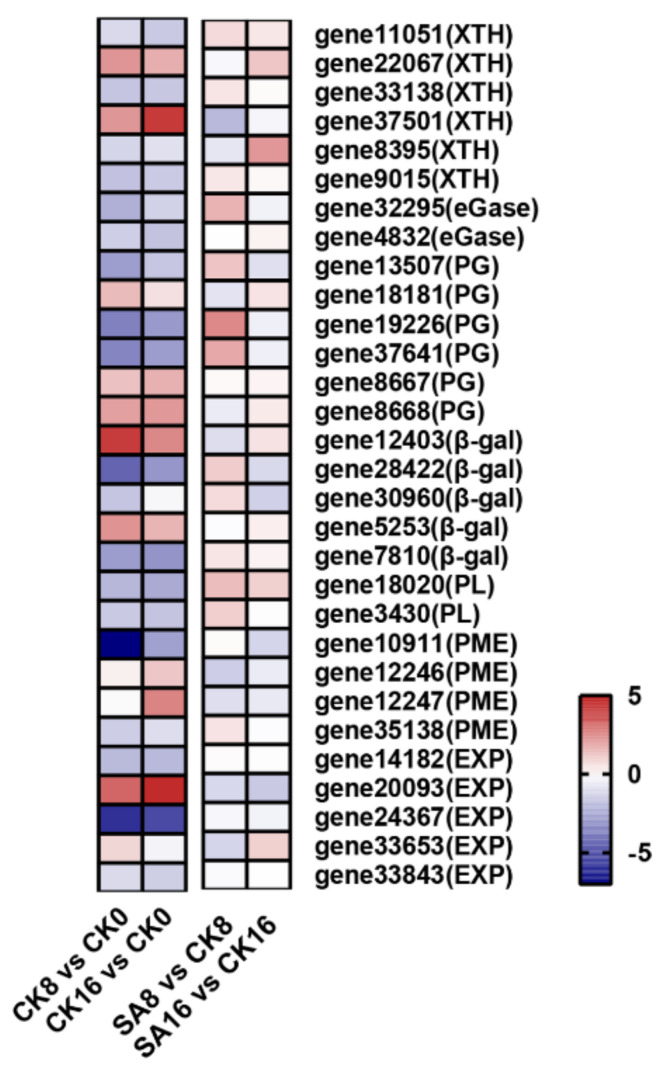
Expression profiles of DEGs associated with cell wall degradation. Gradient colors represent the log_2_ fold change in gene expression between different times and treatments. The red and blue colors indicate the up− and downregulated genes, respectively.

As is known, pear fruit’s postharvest softening is closely related to cell wall degradation (Figure 1B and Figure 2). To investigate the mechanism of exogenous SA regulating pear fruit’s postharvest softening, we further analyzed the DEGs related to cell wall degradation. A total of 30 target genes were identified from RNA-Seq, including xyloglucan endotransglucosylase/hydrolase (XTH), endo-1,4-β-glucanases (eGase), PG, β-gal, PL, PME, and expansin (EXP) (Figure 7 and Table S5). Here, the expression levels of two *XTH* genes (*gene22067* and *gene37501*), three *PG* genes (*gene18181*, *gene8667*, and *gene8668*), two *β-gal* genes (*gene12403* and *gene5253*), and one *EXP* gene (*gene20093*) were apparently upregulated during the storage of the control fruit (Figure 7, Table S5). In comparison to the control pear fruit, exogenous SA treatment obviously inhibited the expression of *gene37501* (encoding XTH), *gene12403* (encoding β-gal), *gene12246* (encoding PME), and *gene20093* and *gene33653* (encoding EXP) (Figure 7 and Table S5).

### 2.8. Differentially Expressed TFs

**Figure 8:**
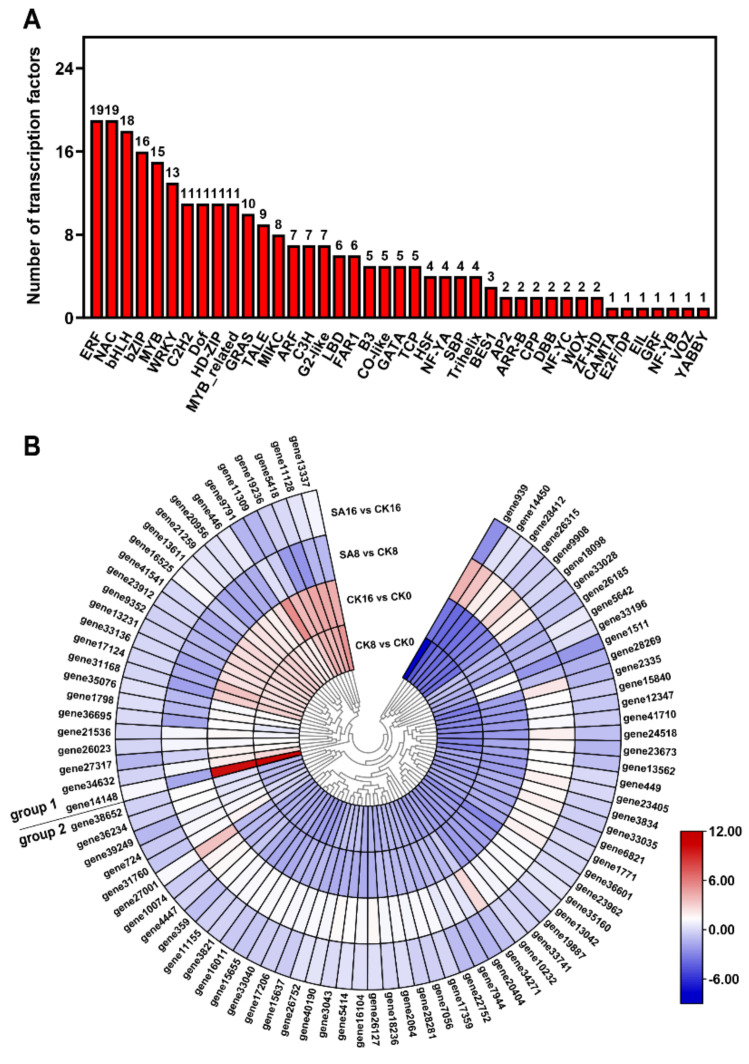
TFs that were differentially expressed during pear fruit ripening and in response to SA treatment: (**A**) The numbers of differentially expressed TFs in different families. (**B**) Expression heatmaps of the DEGs encoding the TFs. Group 1 and group 2 indicate opposite expression patterns of TFs in response to SA treatment. Gradient colors represent the log_2_ fold change in gene expression between different times and treatments. The red and blue colors indicate the up− and downregulated genes, respectively.

To investigate the regulatory networks influencing pear fruit’s ripening and SA response, we detected 264 differentially expressed TFs from 41 TF families, consisting of 235 differentially expressed TFs in the CK8 vs. CK0 and CK16 vs. CK0 comparison groups and 120 differentially expressed TFs in the SA8 vs. CK8 and SA16 vs. CK16 comparison groups (Table S6). The TF families with the most identified DEGs were ERF (19, 7.2%), NAC (19, 7.2%), bHLH (18, 6.8%), bZIP (16, 6%), and MYB (15, 5.7%) (Figure 8A). Meanwhile, eighty-nine TFs were differentially expressed in both the control and SA-treated pear fruits, and we subsequently analyzed their gene expression levels and clustered the genes into two groups: group 1 contained most of the TFs that were upregulated in the CK8 vs. CK0 and CK16 vs. CK0 comparison groups but downregulated in the SA8 vs. CK8 and SA16 vs. CK16 comparison groups; group 2 contained most of the TFs that exhibited the opposite expression pattern compared with group 1 (Figure 8B). The results confirm that a series of differentially expressed TFs are involved in the regulation of pear fruit’s ripening and SA response.

### 2.9. Validation of RNA-Seq by qRT-PCR Analysis

**Figure 9:**
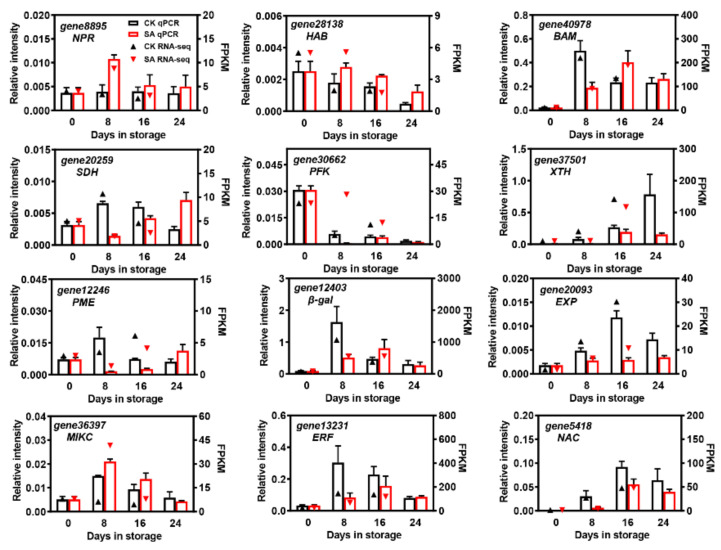
The qRT-PCR validation of DEGs. Relative expression levels from qRT-PCR were calculated using actin as a standard. The columns represent the data obtained from qRT-PCR, while the arrows represent the data obtained from RNA-Seq.

To confirm the reliability of the DEGs identified by RNA-Seq, twelve significantly expressed DEGs that were putatively involved in plant hormone signal transduction, starch and sucrose metabolism, and cell wall degradation, along with three differentially expressed TFs, were selected for qRT-PCR analysis. As shown in Figure 9, most of the DEGs showed significantly different expression profiles between the SA treatment and controls during pear fruit storage. Except for *gene8895* (*non-expresser pathogenesis-related gene* (*NPR*)), *gene28138* (*hypersensitive to ABA* (*HAB*)), and *gene36397* (*MIKC*), the expression of other genes was repressed by SA, especially on the 8th day of room-temperature storage. Moreover, the relative expression levels of *gene37501* (*XTH*), *gene20093* (*EXP*), and *gene5418* (*NAC*) gradually increased in control pear fruit and reached maximal expression after 16 days of room-temperature storage while they remained relatively lower in SA-treated pear fruit (Figure 9). In addition, the relative expression levels of the selected DEGs were consistent with the overall trend results of the transcriptomic datasets, suggesting the high accuracy of the RNA-Seq data (Figure 9).

## 3. Discussion

Rapid postharvest ripening progression in pears strongly shortens their shelf life and restricts their industrial development [1,6,33]. To date, attempts to extend shelf life and reduce quality loss have been widely explored in pears, and several effective techniques, including low temperature, 1-MCP, controlled atmosphere/modified atmosphere packaging, phytohormones, and edible coatings, have been utilized to extend postharvest storability and maintain fruit quality [9,10,11,12,13,14,40]. For example, postharvest fumigation in fruits of several pear cultivars effectively reduced the fruits’ respiration and substantially suppressed their quality deterioration, including acid metabolism, weight loss, flesh firmness, and browning during the post-storage ripening period [4,12,41,42]. Modified atmosphere packaging reduced ethylene production, ascorbic acid degradation, and cell membrane peroxidation and increased the storage life of ‘Doyenne du Comice’ (*P. communis*) pears by up to 2 months [43]. In ‘Kosui’ (*P. pyrifolia*) pear fruits, chitosan/alginate-based layer-by-layer coatings minimized the fruits’ respiration and ethylene production rates, inhibited flesh firmness loss, prevented peel color changes, and prolonged shelf life [44]. As a critical defense-related hormone, SA and its derivatives also show marked effects in delaying the progression of postharvest ripening and extending pear fruit’s shelf life, compared with other storage techniques such as editable coatings [34,35,36,37]. However, the precise mechanism is less known. In the current study, exogenous application of SA to harvested ‘Hosui’ pears significantly delayed their ripening progression, including reducing weight loss, alleviating firmness reduction and cell wall degradation, and retaining higher total phenols simultaneously (Figure 1 and Figure 2), which is consistent with previous studies on postharvest fruits [45,46]. Meanwhile, exogenous application of SA diminished the soluble sugar contents during storage compared with the controls (Figure 3). The effects of SA on soluble sugars vary between different species and cultivars. In pomegranate [47] and peach [48] fruits, postharvest treatment with SA reduced the total soluble solids (TSS) and soluble sugars during storage, while Davarynejad et al. [49] and Wang et al. [32] reported that exogenous SA significantly increased the fruit TSS in plums and kiwifruit.

Subsequent transcriptome analysis showed that there were 3837 and 1387 DEGs identified during room-temperature storage in control fruit and between SA-treated and control fruit, respectively, and many of the selected DEGs (78 DEGs) were significantly enriched in the ‘plant hormone signal transduction’ pathway (Figure 4 and Figure 5). Among them, genes involved in ethylene signal transduction cannot be neglected, as ethylene plays a critical role in climacteric fruit ripening [50]. Here, five ethylene-signal-transduction-related genes (two *ETR*, one *EIN3*, and two *EBF2*) were identified as upregulated during pear fruit ripening, while three of them (one *ETR* and two *EBF2*) were negatively regulated by SA treatment (Figure 5 and Table S3), suggesting their potential vital roles in pear fruit’s ripening and SA response. Moreover, 42 genes (53.8%) involved in the auxin signal transduction pathway were identified as differentially expressed during pear fruit ripening and in response to exogenous SA treatment (Figure 5 and Table S3). Auxin plays an inhibitory role in fruit ripening, and reduced auxin signaling activity could increase the sensitivity of fruit to ethylene [51,52]. In the present study, most of the auxin-signal-transduction-pathway-related genes were downregulated during pear fruit ripening and upregulated in SA-treated pear fruits, and one *AUX/IAA* gene (*gene5126*) was most dramatically changed, implying its potential role in SA-reduced pear fruit ripening (Figure 5 and Table S3). In papaya, the auxin-responsive genes *CpIAA9/17/27* may be candidate genes in fruit ripening [53]. Next to auxin, eight genes involved in the brassinosteroid signal transduction pathway and five genes involved in the cytokinin, abscisic acid, and gibberellin signal transduction pathway were identified as DEGs (Figure 5). These data indicate that the mechanism of SA application delaying ‘Hosui’ pear fruit’s ripening may be through various plant hormones.

Starch and soluble sugars are the primary flavor components of fleshy fruits, which determine their postharvest shelf life and quality [54]. Previous studies have recognized several sugar biosynthesis- and metabolism-related genes that were involved in the postharvest storage of pear fruit, e.g., *PpAc-Inv1*, *PpII5*, *PuAI*, *PuNI*, *PuSS*, and *PuSPS* [23,24,25]. Here, a series of DEGs were also highly enriched in the ‘Starch and sucrose metabolism’ pathway, and the expression levels two *INVs* (*gene22918* and *gene32825*) were highly correlated with the changes in soluble sugars in postharvest ‘Hosui’ fruit (Figure 3, Figure 4 and Figure 6). INV irreversibly hydrolyzes sucrose into glucose and fructose [55,56]. It can be inferred from the results that exogenous SA may inhibit the expression of DEGs encoding INV, which can reduce the soluble sugar contents.

The modification and breakdown of plant cell wall polysaccharides, including cellulose, hemicellulose, and pectin, are responsible for fruit’s softening and textural changes during fruit ripening, and extensive studies have focused on cell-wall-degrading and -modifying enzymes and corresponding genes, which synergistically contribute to the fruit softening phenotype [12,57,58]. In this study, transcriptome data showed that several DEGs associated with cell wall metabolism, including *XTH* (*gene37501*), *β-gal* (*gene12403*), *PME* (*gene20093*), and *EXP* (*gene33653*), were upregulated during the storage of control fruit and downregulated by exogenous SA, indicating their potential roles in the postharvest softening of ‘Hosui’ pear fruit (Figure 7). XTH is involved in hemicellulose metabolism, while β-gal and PME are responsible for pectin modification [58]. In tomato, overexpression or loss-of-function of *SlXTH1* and *SlPMEU1* altered the fruit ripening and softening process [59,60]. The antisense of strawberry *FaβGal4* significantly reduced fruit softening [61]. EXP is involved in cell wall relaxation, and modification of *SlEXP1* in tomato influenced cell wall polymer metabolism during fruit ripening [62].

Fruit-quality-related TFs have been reported in diverse fruit species, and their detailed regulatory effects have also been defined, including for tomato *SlNOR* (NAC TF) in fruit ripening and softening [63], banana *MaDREB2* (ERF TF) and *MabHLH6* in aroma production and starch degradation [64,65], and strawberry *FvMYB10* in anthocyanin accumulation [66]. Here, we identified many TFs that were differentially expressed in the control and SA-treated ‘Hosui’ pear fruits, e.g., *ERF*, *NAC*, *bHLH*, *bZIP,* and *MYB* genes (Figure 8A). These results are consistent with those of previous studies showing that exogenous SA could enhance the antioxidant systems and maintain the storage quality of winter jujube fruit during cold storage by activating many TFs, including the *MYB*, *ERF*, *C2H2 (Cys-2/His-2)*, and *WRKY* genes [67]. Furthermore, different TFs exhibited diverse expression patterns in the control and SA-treated pear fruits, indicating their potential function in ‘Hosui’ pear fruits’ ripening and SA response (Figure 8B).

The present study showed that exogenous SA treatment effectively maintained fruit quality and delayed postharvest ‘Hosui’ pear fruit ripening. Moreover, transcriptome analysis showed that salicylic acid altered the transcripts of the genes related to hormone signaling, sugar metabolism, cell wall modification, and TFs, providing important information for identifying the genes involved in ‘Hosui’ pear fruit ripening. Further functional analyses of these candidate genes will be required to reveal the SA-mediated molecular mechanisms underlying ‘Hosui’ pear fruit ripening. These findings will guide the application of SA in sand pear fruit and could be a helpful reference for fruit preservation technology in food engineering.

## 4. Materials and Methods

### 4.1. Plant Material and Treatments

Commercial mature ‘Hosui’ pear fruits (approximately 150 d after full bloom) were harvested from homogeneous trees in a commercial orchard in Yangzhou (Jiangsu Province, China) in 2022. After transportation to the laboratory, two hundred uniform and defect-free fruits were selected and randomly divided into two groups for the control and SA treatments. SA treatment was applied by immersing the pear fruits for 20 min in a solution of 2 mmol/L SA (analytical reagent, 99.5%, Macklin Biochemical Technology, Shanghai, China), while pear fruits in the control group were immersed in sterile water, and then the fruits were dried in air and held at 20 °C for room-temperature storage. Fruits were sampled at 0, 8, 16, and 24 days, and at each sampling point, fruit flesh from three replicate samples, each of which consisted of four fruits, were sampled for each treatment, cut into small pieces, frozen in liquid nitrogen, and stored at −80 °C for further experiments.

### 4.2. Weight and Firmness

Fifteen fruits for each treatment were weighed at each sampling point. Fruit weight was recorded using a BSA124S-CW balance (Sartorius, Gottingen, Germany).

Fruit firmness was measured with a GY-4 penetrometer (Zhejiang Top Instrument, Hangzhou, China) fitted with an 8 mm probe to puncture the fruit at a 10 mm sample depth and determine the maximum penetration force of the ‘Hosui’ pear fruit after the removal of a small piece of peel. The firmness of each fruit was averaged from two measurements 90° apart at the fruit equator. Fruit firmness was expressed in newtons (N), and 9 individual fruit replicates were used.

### 4.3. Determination of Total Phenols and Ascorbic Acid

Total phenols were determined using the Folin–Ciocâlteu (FC) method [68]. Briefly, frozen fruit flesh samples were thawed overnight at room temperature before drying. The fruit samples were dried using a hot-air dryer set at 65 °C until constant weight and then ground and screened through an 80-mesh sieve. Dried fruit flesh (0.1 g per sample) was ground into a powder, and 2 mL of ethanol (60%) was added to extract the total phenols. The levels of total phenols were calculated from the standard curve drawn using pure gallic acid and were expressed as gallic acid equivalents (GAE)/g dry weight basis. The total phenols of each sample were replicated 3 times (*n* = 3).

The levels of ascorbic acid were determined following the procedure described previously by Hughes [69]. Briefly, 10 g of frozen fruit flesh was homogenized with an equal volume of 2% oxalic acid solution. The filtrate was titrated with 2,6-dichloroindophenol until the color was pale pink and did not fade within 15–30 s. There were 3 replicates for each treatment.

### 4.4. Determination of Cell Wall Components

Cell wall components were extracted and measured as described previously [70,71]. Approximately 1 g of frozen fruit flesh per replicate was washed and centrifuged sequentially with 80% (*v*:*v*) ethanol and acetone. After air-drying the residue at 20 °C for 2 d, the CWM was obtained. Approximately 15 mg of dried CWM was fractionated by extraction with distilled water, 50 mM CDTA and sodium acetate (pH 6.5), and 50 mM Na_2_CO_3_ (including 2 mM CDTA) in sequence to obtain WSP, ISP, and CSP, respectively. Subsequently, the residue was extracted with 4 M KOH at 20 °C for 5 h of continuous shaking, and the supernatant was designated as the hemicellulose content. Finally, the insoluble residue was washed with 0.3 M acetic acid and 80% (*v*/*v*) ethanol and then centrifuged. The pellet was dried at 40 °C and weighed as α-cellulose.

Pectin was determined by the carbazole method [72]. First, 3 mL of H_2_SO_4_ was added to 0.5 mL of extracted solution and boiled for 20 min. After the mixture was cooled down to room temperature, 0.2 mL of carbazole-anhydrous ethanol (1.5 g/L) was added. After 30 min of standing, the absorbance was measured at 530 nm. Hemicellulose and cellulose were extracted and measured according to the anthrone–sulfuric acid method [73]. The extracted cellulose and hemicellulose solutions were each mixed with 5 mL of anthrone reagent (2 g of anthrone dissolved in 80% [*v*/*v*] H_2_SO_4_ and diluted with 80% H_2_SO_4_ to 1000 mL). The mixtures were then heated in a 100 °C water bath for 10 min. After cooling to room temperature, absorbance measurements were carried out at 625 nm. The results were expressed in mg/g. Each component was replicated 3 times (*n* = 3).

### 4.5. Determination of Sugar Components

Soluble sugars, including sucrose, glucose, fructose, and sorbitol, were measured as described by Miao et al. [74], with slight modifications. Briefly, 1 g fruit flesh samples were ground into homogenate with 1 mL of deionized water and centrifuged at 12,000 g for 10 min at 4 °C. Then, the supernatant was passed through a 0.22 mm aqueous membrane filter for further analyses. The levels of individual sugars were determined using high-performance liquid chromatography (HPLC, Agilent 1200-6460 QQQ, Santa Clara, CA, USA) equipped with a carbohydrate analysis column (5 μm, 300 × 6.5 mm, Waters-SugarPak1, Milford, MA, USA). The flow rate of the mobile phase (deionized water) was 0.5 mL/min. Each component was replicated 3 times (*n* = 3).

### 4.6. RNA Extraction and RNA-Seq

The total RNA of ‘Hosui’ pear fruit obtained from each sampling point was extracted by the hexadecyl trimethyl ammonium bromide (CTAB) method as described previously [75]. In order to evaluate the transcriptomic dynamics underlying different treatments, RNA-Seq and bioinformatics analyses were performed using samples (0, 8, and 16 d, including control and SA treatment) with three biological replicates. The experimental parameters were similar to those used in our previous reports [76]. Briefly, RNA-Seq libraries were generated with the NEB Next^®^ Ultra™ RNA Library Prep Kit for Illumina (NEB, Ipswich, MA, USA) and were then sequenced using the Illumina Novaseq6000 platform by Gene Denovo Biotechnology (Guangzhou, China). The clean reads were obtained by removing poor-quality reads (unknown nucleotides > 10% or low Q values ≤ 20%) and mapped to the *Pyrus bretschneideri* ‘DangshanSuli’ genome (version 1.1, https://www.rosaceae.org/species/pyrus_bretschneideri/genome_v1.1, accessed on 15 December 2022). Genes with average FPKM ≥ 1, |log2 (fold change)|≥ 1, and *p*-values < 0.05 were assigned as the DEGs.

### 4.7. cDNA Synthesis and qRT-PCR Analysis

Approximately 1 μg of total RNA was reverse-transcribed into first-strand cDNA using a PrimeScript™ RT reagent kit in conjunction with gDNA Eraser (Takara, Tokyo, Japan). Twelve significantly expressed DEGs were selected for qRT-PCR analysis, and gene-specific primers were designed using the online software Primer3 (the primer sequences are listed in Table S7). Then, qRT-PCR was carried out as described in our previous reports, with the same system and program [76]. The relative expression of the examined genes was normalized using the Ct value corresponding to the pear actin gene (GU830959.1), with three biological replicates.

### 4.8. Statistical Analysis

Data analysis was performed with Microsoft Excel. The significant differences between treatments were identified using two-tailed Student’s *t*-tests. The asterisks indicate significant differences: * *p* < 0.1; ** *p* < 0.01; *** *p* < 0.001. Bar graphs and heatmaps were constructed using GraphPad Prism 7.0 scientific software (San Diego, CA, USA).

## Data Availability

The RNA-Seq datasets generated during the current study have been deposited at NCBI with the project ID PRJNA1002124, and other data supporting the results are included in this published article and its Supplementary Materials.

## Supplementary Materials

The following supporting information can be downloaded at: https://www.mdpi.com/article/10.3390/plants12193429/s1, Table S1: Statistics on the quality and output of the RNA-Seq libraries; Table S2: Differentially expressed genes identified through transcriptome analysis; Table S3: Differentially expressed genes involved in pathways related to plant hormone signaling transduction; Table S4: Differentially expressed genes involved in starch and sucrose metabolism pathways; Table S5: Differentially expressed genes involved in cell wall metabolism; Table S6: Differentially expressed transcription factors between the control and SA-treated pear fruits during postharvest storage; Table S7: Primers used for qRT-PCR analysis in this study.
